# The Relationship between Leptin, the Leptin Receptor and FGFR1 in Primary Human Breast Tumors

**DOI:** 10.3390/cells9102224

**Published:** 2020-10-01

**Authors:** Wyatt Boothby-Shoemaker, Vanessa Benham, Shreya Paithankar, Rama Shankar, Bin Chen, Jamie J. Bernard

**Affiliations:** 1 Department of Pharmacology and Toxicology, Michigan State University, East Lansing, MI 48824, USA; boothbys@msu.edu (W.B.-S.); benhamva@msu.edu (V.B.); chenbi12@msu.edu (B.C.); 2 Department of Pediatrics and Human Development, Michigan State University, Grand Rapids, MI 49503, USA; paithank@msu.edu (S.P.); ramashan@msu.edu (R.S.); 3 Nicolas V. Perricone Division of Dermatology, Department of Medicine, Michigan State University, East Lansing, MI 48824, USA

**Keywords:** FGF2, leptin, FGFR1, leptin receptor, body mass index, obesity, overweight, body mass index, body fatness

## Abstract

Obesity is associated with increased breast cancer risk and poorer cancer outcomes; however, the precise etiology of these observations has not been fully identified. Our previous research suggests that adipose tissue-derived fibroblast growth factor-2 (FGF2) promotes the malignant transformation of epithelial cells through the activation of fibroblast growth factor receptor-1 (FGFR1). FGF2 is increased in the context of obesity, and increased sera levels have been associated with endocrine-resistant breast cancer. Leptin is a marker of obesity and promotes breast carcinogenesis through several mechanisms. In this study, we leverage public gene expression datasets to evaluate the associations between FGFR1, leptin, and the leptin receptor (LepR) in breast cancer. We show a positive association between FGFR1 and leptin protein copy number in primary breast tumors. These observations coincided with a positive association between Janus kinase 2 (Jak2) mRNA with both leptin receptor (LepR) mRNA and FGFR1 mRNA. Moreover, two separate Jak2 inhibitors attenuated both leptin+FGF2-stimulated and mouse adipose tissue-stimulated MCF-10A transformation. These results demonstrate how elevated sera FGF2 and leptin in obese patients may promote cancer progression in tumors that express elevated FGFR1 and LepR through Jak2 signaling. Therefore, Jak2 is a potential therapeutic target for FGFR1 amplified breast cancer, especially in the context of obesity.

## 1. Introduction

Obesity, as defined by the body mass index (BMI) > 30 kg/m^2^, is increasing globally and contributes to both cancer risk and mortality worldwide representing an enormous cost to public health [1,2,3,4,5]. One hundred million children and adolescents and 600 million adults are currently obese [1,2] and constitute an at-risk segment of the population for developing obesity-linked malignancies, such as breast cancer. An elevated BMI is associated primarily with postmenopausal, estrogen receptor (ER)-positive (ER^+^) breast cancer; however, in pre-menopausal women, obesity is associated with a lower risk of ER^+^ breast cancer and a higher risk of triple negative breast cancer (TNBC) [6,7,8,9,10,11]. Moreover, central/visceral obesity, which reflects the accumulation of a particularly harmful type of adipose tissue, visceral adipose tissue (VAT), is a strong predictor of premenopausal TNBC [6,9]. The molecular mechanisms underlying breast cancer risk with both general/overall and central/visceral adiposity are complex and have not been fully elucidated.

One of the suspected pathophysiological mechanisms by which excess adiposity promotes breast cancer is through the release of leptin from adipocytes. Leptin plasma levels increase in proportion to overall obesity [12]. Research indicates that leptin is produced and secreted from VAT, but the relative contribution of circulating leptin to the local release of leptin from the mammary adipose tissue or to tumor progression is unknown. Leptin activates signaling pathways through the transmembrane leptin receptor and is involved in cell proliferation and invasion. Specifically, leptin is thought to promote breast cancer through the activation of Janus kinase 2/Signal Transducers and Activators of Transcription 3 (Jak2/Stat3), mitogen-activated protein kinase (MAPK) and Phosphatidylinositol-3-kinase/Protein kinase B (PI3K-AKT) [13]. Leptin-stimulated tumor growth is not limited to breast cancer, but is to melanoma [14] and prostate cancer [15] and several other obesity-linked cancer types recently reviewed by Modzelewska and colleagues [16].

Adipose tissue-derived fibroblast growth factor-2 (FGF2) may constitute an additional marker of breast cancer risk, particularly in the context of visceral adiposity. FGF2 is a growth factor that drives proliferation, angiogenesis and development, primarily through fibroblast growth factor receptor-1 (FGFR1). In breasts, FGF2 and FGFR1 are physiologically important in mammary duct development [17]. As circulating FGF2 levels correlate with adipose tissue mass in humans, FGF2/FGFR1 signaling could be a potential mechanism in which obesity influences the mammary gland [18]. Interestingly, our previous data demonstrate that removing parametrial adipose tissue (the largest visceral fat pad in female mice) by lipectomy in mice fed a high-fat diet (HFD), significantly reduces serum FGF2 [19]. While traditionally thought to be a paracrine acting growth factor, these data suggest that FGF2 may be released from adipose tissue and act via endocrine mechanisms. Additionally, we have demonstrated that FGF2 levels are positively associated with the neoplastic transforming capacity of human adipose tissue on mouse mammary epithelial cells, as measured by anchorage-independent growth in soft agar [20]. We also demonstrated that the activity of adipose tissue on human mammary epithelial cell (MCF-10A) transformation is dependent on FGFR1 activity [21]. These data demonstrate a relationship between FGF2/FGFR1 signaling and malignant transformation in the context of adiposity and breast cancer, in vitro.

While FGF2 and leptin are both physiologically relevant in mammary gland development, we hypothesize that higher than normal circulating levels of these adipokines may be contributory to breast cancer in the context of obesity, especially for tumors with FGFR1 amplifications and increased LepR expression. Independently, high FGFR1 protein expression or high LepR protein expression is implicated in the poor prognosis of breast cancer patients [22,23,24]. Herein, we demonstrate a relationship between FGFR1, leptin, and LepR in human breast tumors. We also demonstrate that treating non-tumorigenic MCF-10A cells with a combination of FGF2 and leptin significantly stimulated transformation (growth in soft agar) as compared to cells treated with FGF2 or leptin individually. Additionally, this FGF2+leptin- or adipose tissue-stimulated growth in soft agar was inhibited with the Jak inhibitors, AG490 and Ruxolitinib. These studies suggest a relationship between FGFR1 and leptin/LepR in breast tumors that may drive tumorigenesis and breast cancer progression in obesity.

## 2. Materials and Methods

### 2.1. Cell Culture

MCF-10A cells (human mammary epithelial cells) were obtained from ATCC (Manassas, VA, USA). Cells were cultured in DMEM/Ham’s F12 media supplemented with 5% horse serum (HS), 1% penicillin/streptomycin, 100 ng/mL cholera toxin, 20 ng/mL epidermal growth factor (EGF), 10 µg/mL insulin, 0.5 mg/mL hydrocortisone, 7.5% sodium bicarbonate, 15mM HEPES, and 2 mM L-Glutamine (growth media). MCF-10A cells were trypsinized with 0.05% trypsin and quenched in DMEM/Ham’s F12 media with 20% horse serum and antibiotics (resuspension media). Mycoplasmas were tested for in culture every three months.

### 2.2. Preparation of Fat Tissue Filtrates

Mouse parametrial adipose tissue was pooled from five SKH1-E mice fed a HFD for 4 weeks. In total, 100 mg of parametrial adipose tissue was gently homogenized in an equal volume of serum-free MEM on ice for 30 s using Tissue Ruptor (Qiagen Hilden, Germany) at medium speed. Homogenates were filtered through a hanging 15 mm-wide 0.4 µm filter insert (Millicell, cat# MCHT06H48, Millipore Sigma, Burlington, MA, USA) into a six-well plate previously filled with 400 µl serum-free MEM and incubated on a rocker at RT for 1 h to allow small molecules and proteins to diffuse into the medium while removing lipids and macromolecules. After, incubation filtrates were centrifuged at 4500 rpm for 5 min and the supernatant was collected and filtered through a 0.4 μm syringe filter (Millipore Sigma) and protein concentrations were quantified using BCA assay. A total of 200 µg/mL of mouse fat tissue filtrate (MFTF) was used.

### 2.3. Anchorage-Independent Colony Formation Soft Agar Assay

MCF-10A cells were seeded at 750 cells per well in a 24-well plate in 200 µL of DMEM/Ham’s F12, 5% HS, and 0.33% agar with or without MFTF and/or inhibitors which was overlaid onto 350 µL of DMEM/Ham’s F12, 5% HS, and 0.5% agar. Soft agar plates were left at room temperature for 30 min before 200 µL of growth media was gently added to each well and then stored at 37°C. Every 3–4 days, the growth media was removed from each well and replenished with 200 µL of growth media. After two weeks, the colonies were fixed in 70% ethanol (EtOH) and stained with 150 µL of 0.01% crystal violet. Colonies were visually counted and used to calculate the percent of colony formation from the number of cells plated ([Colonies counted × 100] / 750 cells). The percentage colony formation was normalized to the untreated control to determine the increase in percentage of colony formation (percentage colony formation of treatment—the percentage colony formation of untreated control). Colonies were quantified on the Cytation 3 imaging reader from Biotek using Gen5 3.04 software (Winooski, VT, USA). Seven pictures were taken every 100 microns and superimposed together by the software’s Z-projection function. The percent of colony formation from the number of cells plated ([Colonies counted × 100] / number of cells plated) were analyzed. Three technical and 2 biological replicates were performed for the soft agar assay.

### 2.4. Western Blotting

A total of 1.4 × 105 cells were plated in 35-mm culture dishes and allowed to grow for 24 h before treatment. The cells were treated with MFTF 200 µg/mL for 2, 8, and 24 h time points. After treatment, cells were collected, washed and lysed -in RIPA buffer pH 7.4 and supplemented with protease and phosphatase inhibitors. Proteins were separated by a 4% to 20% gradient SDS-PAGE gel and transferred to nitrocellulose membranes. Membranes were blocked with either a 5% nonfat milk solution or 5% BSA and then incubated with Rabbit anti-pJak2 antibody (Cell Signaling Technology Cat# 3771; Danvers, MA, USA) and Rabbit anti-Actin loading control antibody (Sigma Cat# A5060) overnight at 4 °C, followed by 1 h incubation with fluorochrome-tagged secondary antibody (LiCor Cat# 926-32213). Bands were visualized by a LI-COR Odyssey classic image scanner (Lincoln, NE, USA).

### 2.5. Statistics

In soft agar experiments, three technical replicates were used to ensure adequate power to detect a significant change in growth in soft agar. Statistical analyses between soft agar treatment groups were evaluated using one-way ANOVAs and one-way unpaired t-tests, with *p*-values being adjusted for multiple comparisons in post-hoc analyses. Soft agar data are presented as mean ± s.e. For all statistical tests, a 0.05 and 0.01 level of confidence, were accepted for statistical significance. For bioinformatics data, cBioPortal’s TCGA Firehouse Legacy database was used to analyze gene sequencing in breast cancer tumors, with the search terms being “FGFR1” and “leptin”. Genomic data generated from cBioPortal represent z-scores and are normalized. A student’s t-test assuming equal variance was performed to distinguish the average differences in copy numbers from cBioPortal. Additional transcriptomic analyses utilized the Spearman correlation to assess the leptin receptor-FGFR1 relationship and log transcript count per million (TPM) in an effort to normalize data. R and Microsoft Excel (Seattle, USA) and Prism (San Diego, USA) software platforms were used for statistical analyses.

## 3. Results

Our results have a bioinformatics-based component and an in vitro-based component to gain insight into the interplay between FGFR1, leptin, and adiposity in breast cancer. Our bioinformatics results analyze publicly available genomic data to describe the expression of FGFR1 and leptin in breast tumors.

### 3.1. Positive Correlation between FGFR1 mRNA and Leptin mRNA in Primary Breast Cancer 

We set out to determine the average mRNA expression of FGFR1, leptin, and LepR in normal breast tissue and breast cancer tissue using publicly available genomic datasets including The Cancer Genome Atlas (TCGA), Tumor Alterations Relevant for Genomics-driven Therapy (TARGET), the Genome-Tissue Expression project (GTEx), and Met500. In addition, we examined significant relationships between the mRNA co-expression of FGFR1 and LepR in 29 primary cancers (Table 1). Breast cancer has the seventh strongest correlation (Rho = 0.177) between average FGFR1 mRNA and average leptin mRNA among all primary cancers that showed the highest significance between FGFR1 mRNA and leptin mRNA (*p* = 3.17 × 10^−9^). Average leptin mRNA expression (0.6713) was the highest in breast cancer compared to other cancer types analyzed (Table 1). Of the 29 types of primary cancer, the average FGFR1 mRNA expression in breast cancer is the sixth highest overall (2.8852), and third highest among cancers where a significant relationship between leptin mRNA and FGFR1 mRNA was observed. These data demonstrate a significant positive correlation between FGFR1 mRNA and leptin mRNA in primary breast cancer and suggest that receptor expression and/or functional synergy may have relevance for tumor progression.

### 3.2. Relationship between Leptin and FGFR1 mRNA in Normal Breast Tissue, Primary Breast Cancer Tumor Tissue and Breast Cancer-Adjacent Tissue

Following the analysis of FGFR1 mRNA and leptin mRNA in primary breast cancer, we analyzed the relationship of FGFR1 mRNA and leptin mRNA in normal breast tissue, breast tumor-adjacent tissue, and tissue from metastatic breast cancer (Table 2). There is a significant relationship between leptin mRNA and FGFR1 mRNA in normal breast tissue, primary breast cancer tumor tissue, and breast cancer-adjacent tissue; however, there is a difference in the directionality of the relationship (Table 2). Associations between leptin mRNA and FGFR1 mRNA expression are positive in breast tumor-adjacent tissue (Rho = 0.26) and primary breast cancer tissue (Rho = 0.16), and negative in normal breast tissue (Rho = −0.27). Despite the low correlation (0.177) and large number of observed associations that may influence this observation towards significance, there is a change between a negative relationship between leptin and FGFR1 mRNA in normal tissue (−0.27) to a positive relationship between average leptin mRNA and FGFR1 mRNA in breast cancer tissue (0.16). No significant associations are found in metastatic breast cancer (Table 2). Interestingly, leptin mRNA is highest for normal tissue and tumor tissue adjacent to breast cancer, but leptin is lower in primary cancer, and the lowest in metastatic cancer (Table 2). These data demonstrate a positive and significant correlation between leptin mRNA and FGFR1 mRNA in both primary breast tumors and the tumor microenvironment.

### 3.3. Leptin Levels in FGFR1 Amplification, Gain, and Diploid ER^-^ Tumors Compared to ER^+^ Breast Tumors

Next, we compared the relationship between leptin mRNA and FGFR1 mRNA among primary breast tumors divided into subgroups according to FGFR1 copy-number level per gene expression by the Genomic Identification of Significant Targets in Cancer (GISTIC). Subgroups were designated as FGFR1 amplification, gain, diploid, shallow deletion, and deep deletion according to the copy number variation. We demonstrated that an increased FGFR1 copy number variation was significantly correlated with an increased leptin copy number in both ER^+^ breast tumors (7.8 × 10^−5^, beta coefficient 0.0367) and ER^-^ breast tumors (6.5 × 10^−7^, beta coefficient 0.1798) (Table 3). When comparing the average values of GISTIC-designated FGFR1 categories, FGFR1 amplification is associated with a significantly higher average relative linear copy number of leptin compared with the average relative linear copy number of FGFR1 diploid, shallow deletion, and deep deletion breast tumors (*p* < 0.05). However, the low number of FGFR1 deep deletion breast tumors available for analysis (*n* = 4) should be noted. When these subgroups were further divided and analyzed according to ER status, we found similar positive associations between the FGFR copy number and leptin mRNA in both ER^+^ and ER^-^ tumors. Interestingly, significantly higher leptin levels were observed in FGFR1 amplification, gain, and diploid ER^-^ tumors compared with these copy number states in ER^+^ breast tumors (*p* < 0.05) (Table 4). These data demonstrate a positive association between leptin and FGFR1 in breast cancer that may be strengthened by the loss of the estrogen receptors in tumors.

### 3.4. LepR mRNA and FGFR1 mRNA Were Positively Correlated in Breast Tissue

**Figure 1:**
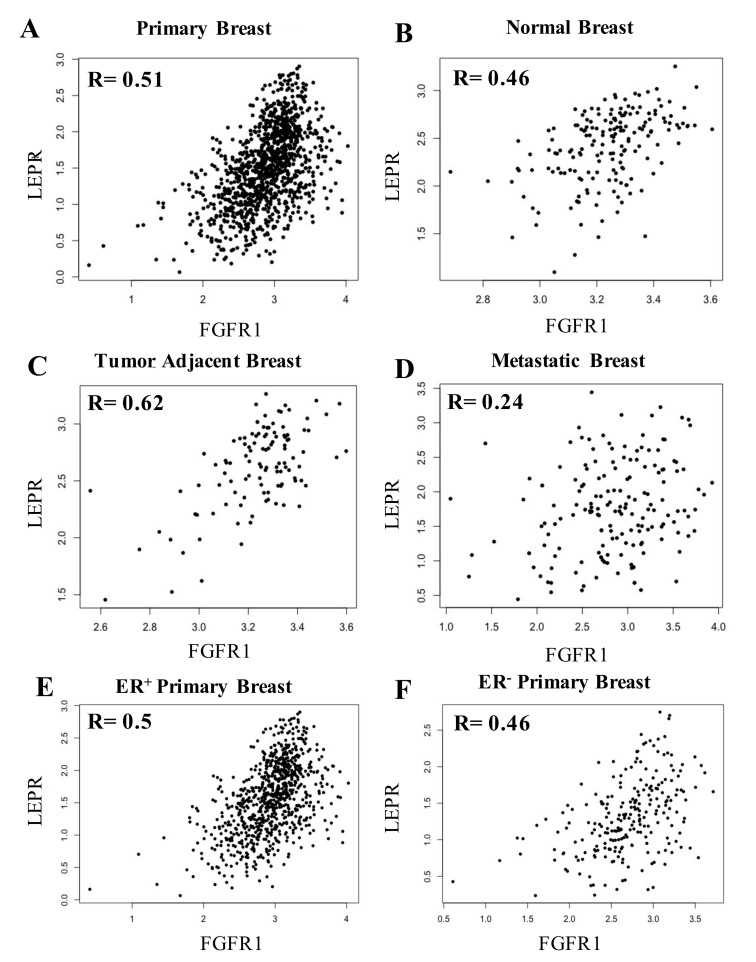
Correlation of expression level of FGFR1 with leptin receptor (LepR) in breast cancer tissue. Expression of FGFR1 with LepR showed a positive strong correlation in breast (**A**) primary tumor tissue (*R* = 0.51), (**B**) normal tissue (R = 0.46) and (**C**) adjacent tissue (*R* = 0.62) but weak association in (**D**) metastatic tissue (*R* = 0.24). In addition, the (**E**) ER^+^ primary tumors showed a stronger positive correlation of FGFR1 with LepR (*R* = 0.5) as compared to (**F**) ER^−^ primary tumors (*R* = 0.46).

These new findings, in conjunction with prior studies demonstrating that leptin drives obesity-mediated breast tumor progression, provided rationale for analyzing the relationship between FGFR1 mRNA and the leptin receptor (LepR) mRNA in breast cancer [25]. Understanding this relationship may provide hypothesis-generating information on a tumor signature that could be sensitive to circulating or a local release of adipokines. In additional breast cancer analyses, RNA-Seq samples were collected from TCGA and processed using an in-house pipeline [26]. Elevated LepR mRNA and FGFR1 mRNA were positively correlated in primary breast tissue (*R* = 0.51), in areas adjacent to the tumor (*R* = 0.62), and to a lesser extent in normal tissue (*R* = 0.46) (Figure 1A–C). A weaker association was observed in metastatic tumors (*R* = 0.24) (Figure 1D). The positive association in primary breast tissue was similar when patients were stratified based on their subtypes (ER^+^, *R* = 0.50; ER^−^, *R* = 0.46) (Figure 1E–F). Collectively, these data demonstrate a positive association between FGFR1, leptin, and the LepR in human primary breast cancer and a much weaker association in metastatic tumors.

### 3.5. Role of Jak2 Activation in Adipokine-Stimulated Mammary Epithelial Cell Transformation

**Figure 2:**
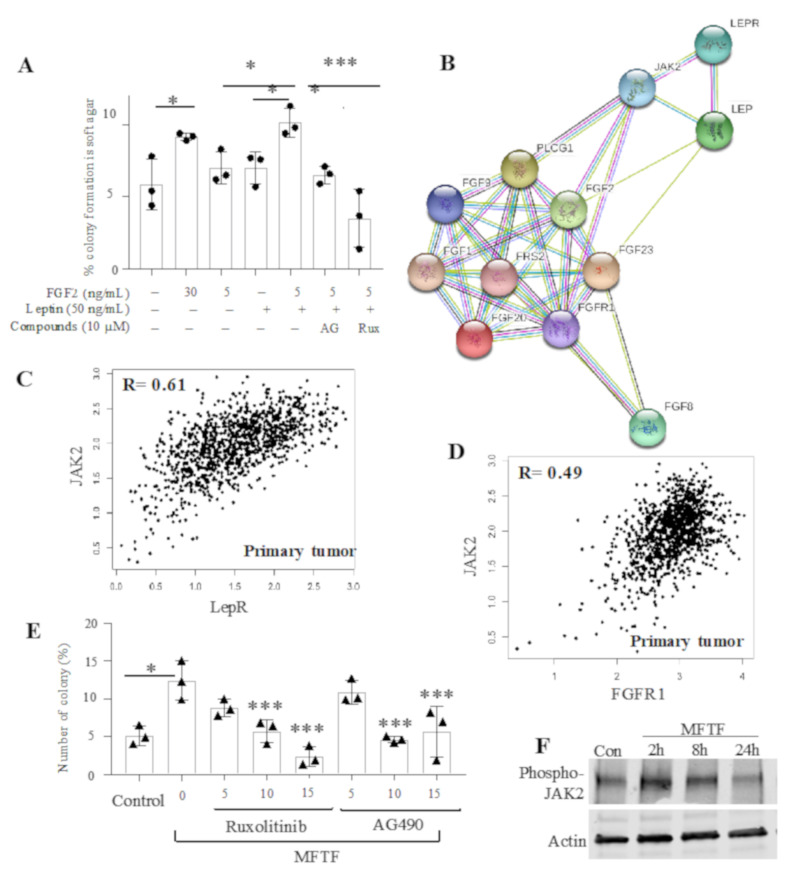
Validation of FGF2 and leptin and their inhibitory effect due to Jak2 inhibitors. (**A**) MCF-10A cells grown in presence of different concentrations of FGF2 and leptin showed enhanced growth when combined. (**B**) Protein network analysis performed between leptin receptor and FGFR1 demonstrate that both proteins are implicated in activating the Jak2 pathway. In primary breast cancer, (**C**) leptin receptor (LEPR) shows a correlation of 0.61 with Jak2 mRNA and (**D**) FGFR1 shows a correlation of 0.49 with Jak2 mRNA. (**E**) MCF-10A cells were treated with 200 µg/mL of mouse fat tissue filtrate (MFTF), treated with Ruxolitinib and AG490, and showed decreased average epithelial transformation in a dose dependent manner. (**F**) MCF-10A cells were treated with 200 mg/mL of MFTF for 2, 8 or 24 h and protein was probed with an anti-phospho-JAK2 antibody. MFTF stimulates JAK2 phosphorylation at 2 h. STRING DB (https://string-db.org/) was used to perform protein network analysis. PLCG1: Phospholipase C gamma, FGF2: Fibroblast growth factor, LEP: Leptin, LEPR: Leptin receptor, FRS2: Fibroblast growth factor receptor substrate 2, Jak2: Janus kinase 2. * *p* < 0.05, *** *p* < 0.001. FGF = Fibroblast growth factor. PLCG1 = Phospholipase C Gamma 1.

The relationship between FGFR1 and LepR in ER^-^ tumors led us to investigate the effect of both FGF2 and leptin on colony formation in human ER^-^ mammary epithelial cells (MCF-10A). We previously demonstrated that FGF2 from adipose tissue stimulates MCF-10A cell growth in soft agar [21]. In this assay, anchorage-independent three-dimensional (3D) growth (colony formation) is a surrogate marker for neoplastic transformation. MCF-10A cells are non-tumorigenic and have a low rate of spontaneous transformation (5%) compared with tumorigenic epithelial cells and express both LepR and FGFR1. MCF-10A cells were treated with 50 ng/mL of leptin and/or 5 ng/mL of FGF2 to simulate the physiologic circulating concentrations of these growth factors in obesity [18]. Colonies (eight cells or greater) were measured and automatically counted using a 3D photographic analysis and the number of colonies was related to the number of cells plated (1000 cells/well) to calculate the percentage of colony formation. Cells treated with FGF2 showed significantly more colony formation compared with untreated cells (Figure 2A). Cells treated with leptin alone failed to show a significant increase in colony formation compared to untreated cells; however, leptin significantly enhanced the effects of FGF2 on colony formation (Figure 2A).

FGFR1 and Jak2 have both been observed to be upregulated in breast cancer [22,27]. To determine potential target pathways that could prevent FGF2+leptin-stimulated transformation, protein-interaction network analysis was performed between LepR and FGFR1, demonstrating that both FGFR1 and leptin converge in activating the Jak2 pathway (Figure 2B). Furthermore, in primary breast cancer, Jak2 mRNA has a positive association with both LepR (*R* = 0.61; Figure 2C) and FGFR1 mRNA (*R* = 0.49; Figure 2D). In fact, MCF-10A cells treated with 200 µg/mL of MFTF for 2 or 8 show and induction of phospho-Jak2 that returns to control levels at 24 h. These data suggest a role for Jak2 activation in adipokine-stimulated tumor promotion.

Since these data (Figure 2B–D) demonstrated Jak2 as a potential link between LepR and FGFR1 activation, MCF-10A cells treated with both FGF2 and leptin were cultured in the presence of the Jak inhibitors AG490 and Ruxolitinib. AG490 is a tyrosine kinase inhibitor that inhibits the Jak2 pathway in breast cancer cells in vitro by decreasing the phosphorylation of Stat3 [25]. Ruxolitinib is a tyrosine kinase inhibitor that selectively inhibits Jak1 and Jak2 signaling and is being evaluated in multiple breast cancer clinical trials for its chemotherapeutic effects [28,29]. Both AG490 (10 µM) and Ruxolitinib (10 µM) significantly inhibited FGF2+leptin-stimulated colony formation (Figure 2A). AG490 and Ruxolitinib were further tested for their ability to inhibit mouse fat tissue filtrate (MFTF)-stimulated MCF-10A cell transformation. MFTF was generated from the parametrial adipose tissue of high-fat diet (HFD)-fed mice as previously described [19,21,30]. MCF-10A treatment with MFTF showed a significantly higher number of colonies compared with no treatment. AG490 and Ruxolitinib both attenuated MFTF-stimulated cell transformation in a concentration-dependent manner (Figure 2E).

## 4. Discussion

The relationship between breast cancer and obesity is complex and is influenced by tumor subtype, menopausal status and adipose tissue distribution. An elevated BMI is associated primarily with postmenopausal, ER^+^ breast cancer [31] but is protective in premenopausal breast cancer [31] with one critical exception: visceral adipose tissue accumulation in premenopausal breast cancer is a risk factor for triple negative breast cancer (TNBC) [6,8,9,10,11] and HER2**^+^**ER^−^ [8]. The precise mechanisms behind these complexities are not fully understood. While the studies herein do not fully elucidate these complexities, they provide relationship data on two receptors in breast tumors that may be influenced by excess adipose tissue and drive tumor progression, LepR and FGFR1. Herein, we found that, of all primary tumors in 29 different tissues, primary breast tumors demonstrated the most significant relationship between leptin mRNA and FGFR1 mRNA and had the highest levels of average leptin mRNA among other primary cancers of comparison. Both FGFR1 and LepR, independently, have been shown to increase breast tumor size and lead to a reduced overall survival in breast cancer patients [22]. One report demonstrated that a high expression of LepR in breast cancer tissue predicts poorer outcomes in patients with high, but not low, sera leptin [24]. FGFR1 amplifications have been reported to be present in 10% of all breast cancers [32] and increased protein expression of FGFR1 is associated with having a poorer clinical prognosis [32], and greater tumor size [33]. Therefore, a high FGFR1/leptin/FGFR1 breast tumor signature may be most detrimental when circulating levels of FGFs and leptin are elevated.

In both ER^+^ and ER^−^ breast cancers, there was a positive association between FGFR1 mRNA, and LepR mRNA, although ER^-^ tumors had significantly higher leptin mRNA compared to ER^+^ tumors. Without estrogen signaling, it is attractive to speculate that tumor progression may be driven by other signaling factors such as either FGFR1 signaling and/or LepR signaling. Emerging studies are in support of this idea. Wellberg et al. demonstrated that FGFR1 activation promotes mammary tumorigenesis in an in vivo model of obesity in which tumors became resistance to endocrine therapy. Additionally, an ER^-^ breast cancer cell line was shown to maintain paracrine loop signaling from 17β-estradiol and induce the upregulation and secretion of FGF2 and increase FGFR1 signaling [34]. In another study, FGFR1-amplified tumors had increased expression of genes integral to cell cycle progression in ER^+^ endocrine-resistant breast cancer, and suggest that FGFR1 amplification promotes cancer cell growth and endocrine resistance [35]. Leptin and its effects on the progression of breast cancer have also been implicated in the development of endocrine resistance. LepR downregulation was shown to increase sensitivity to Tamoxifen [36]. Moreover, it has been proposed that higher LepR mRNA expression and leptin signaling genes had a more fatal impact on ER^-^ subtypes compared with ER^+^ breast cancer [37].

Due to the poor clinical implications of FGFR1 amplification and LepR status, it would be beneficial to identify functional crosstalk between amplified FGFR1 and leptin signaling. Our protein-interaction network analysis demonstrated functional crosstalk between the activation of these two receptors and Jak2 and our Western blot analysis demonstrates that MFTF stimulates the phosphorylation of Jak2. Jak2 activation has been shown to be the primary pathway by which the LepR functions on the neuroendocrine system [38] and STAT3 signaling has been implicated in promoting obesity-mediated cancer progression [25]. We demonstrated that both LepR mRNA and FGFR1 mRNA were both positively associated with Jak2 mRNA in primary breast tumors. To determine if Jak2 inhibitors had therapeutic efficacy in response to FGF2 and leptin-driven transformation we treated ER^-^ MCF-10A cells with FGF2, leptin or in combination. Interestingly, only the combination of FGF2 and leptin significantly stimulated MCF-10A growth in soft agar (colony formation). This demonstrates that leptin/LepR and FGFR1 signaling can promote estrogen-independent malignant transformation. Culturing MCF-10A cells in the presence of the Jak inhibitors, AG490 and Ruxolitinib significantly reduced either leptin+FGF2-stimulated colony formation, or MFTF-stimulated colony formation. 

The major limitation of our study is that obesity measurements in the analyzed patients are unknown. Therefore, future studies will involve the recruitment of healthy and cancer subjects to measure body fatness parameters such as BMI and waist circumference. We would also measure FGFR1, LepR, and phospho-Jak2 protein expression in tumor tissue since one shortcoming of our study is that many of our correlations involve mRNA. mRNA levels do not always correspond with protein and their associated activities. It would be of interest to determine in the future how levels of these proteins and Jak/Stat pathway activation markers correspond to tumor subtypes and body fatness.

In conclusion, we define a positive relationship between leptin, LepR and FGFR1 in primary human breast tumors and a positive relationship between LepR and FGFR1 in the tissue adjacent to tumors. Crosstalk between these two signaling pathways may occur through Jak2. Consequently, Jak2 inhibitors may be a useful therapeutic target for the treatment of tumors expressing high FGFR1 and LepR when patients have high sera FGF2 and leptin. Future studies will be aimed determining the exact contribution of FGF2 and leptin to early-stage breast carcinogenesis and progression. Of particular interest is how these molecular mechanisms underlie breast cancer risk based on subtypes with both general/overall and central/visceral adiposity to help unravel the complex relationship between obesity and breast cancer.

## Figures and Tables

**Table 1:** Comparing FGFR1 mRNA and Leptin mRNA by Primary Cancer.

Primary Cancer Type	Rho	*p*-Value	Samples	Average Leptin mRNA	Average FGFR1 mRNA
White Blood Cell	0.3421	4.08×10^−6^	200	0.1364	2.3125
Colon	0.2846	8.32×10^−7^	463	0.1169	2.1402
Pancreas	0.2837	0.0001	186	0.4168	2.8082
Rectum	0.2665	0.0098	168	0.1098	2.2070
Bile Duct	0.2494	0.1424	36	0.0294	2.8067
Testis	0.2363	0.0032	156	0.3647	3.0769
Stomach	0.1915	8.83×10^−5^	478	0.1513	2.4725
Breast	0.1774	3.17E-09	1108	0.6713	2.8852
Bladder	0.1736	0.0004	413	0.2391	2.0195
Ovary	0.1450	0.0027	613	0.1288	2.8148
Skin	0.1408	0.0022	478	0.1434	2.6506
Thyroid Gland	0.1405	0.0014	515	0.0528	2.4212
Body Cavity	0.1350	0.2126	87	0.1555	3.0533
Liver	0.1177	0.0234	379	0.0363	1.4615
Lung	0.1138	0.0003	1091	0.2550	2.5369
Eye	0.1073	0.3464	80	0.0082	2.1440
Brain	0.0878	0.0211	1138	0.0274	2.8951
Adrenal Gland	0.0866	0.4539	92	0.0539	2.5385
Bone	0.0661	0.2867	265	0.1456	3.2758
Prostate	0.0615	0.1716	499	0.0247	2.5691
Lymphatic Tissue	0.0540	0.7183	48	0.3418	2.1315
Esophagus	0.0238	0.7500	186	0.1591	2.5131
Thymus	0.0193	0.8350	124	0.0940	2.3847
Uterus	0.0130	0.9237	57	0.0749	2.9587
Paraganglia	−0.0053	0.9435	184	0.0781	3.0194
Kidney	−0.0084	0.8023	895	0.1258	2.7034
Head and Neck	−0.0190	0.6657	530	0.2131	2.2329
Cervix	−0.0498	0.3856	310	0.2104	2.0481
Endometrium	−0.0903	0.2268	548	0.0413	2.6931

mRNA of FGFR1 and leptin from primary tumor samples were compared by primary cancer type in The Cancer Genome Atlas (TCGA) database. A total of 14 of 29 primary cancers showed a significant relationship (*p* < 0.05) between FGFR1 mRNA and leptin mRNA. Breast cancer had the seventh highest association (Rho = 0.1774) and most significant value (*p*-value = 3.17 × 10^−9^).

**Table 2:** FGFR1 mRNA and Leptin mRNA in Breast Tissue.

Breast Tissue Type	Rho	*p*-Value	Average Leptin mRNA	Average FGFR1 mRNA
Normal	−0.27	0.0002	3.02 ± 0.09	2.16 ± 0.97
Primary Cancer	0.16	1.36 × 10^-7^	2.63 ± 0.32	0.63 ± 0.71
Breast Tumor Adjacent	0.26	0.0059	2.94 ± 0.1	2.55 ± 0.77
Metastatic Cancer	−0.01	0.97	2.63 ± 0.35	0.38 ± 0.65

In primary breast cancer, average expression of FGFR1 mRNA is most positively correlated with average leptin mRNA in breast tumor-adjacent tissue (Rho = 0.2868), followed by primary cancer tissue (Rho = 0.1774). There is a negative correlation between leptin mRNA and FGFR1 mRNA in normal breast tissue (Rho = −0.2379). There is not a significant correlation between average FGFR1 expression and average leptin mRNA expression in metastatic breast cancer tissue. mRNA samples are expressed in logarithmic format. Samples are taken from TCGA. Pearson correlations were used for computation.

**Table 3:** FGFR1 and Leptin Average Relative Copy Number in Breast Tumors, with ER-Status.

Breast Tumor FGFR1 Copy Number Status	FGFR1 Amplification	FGFR1 Gain	FGFR1 Diploid	FGFR1 Shallow Deletion	FGFR1 Deep Deletion
Average leptin relative copy number	0.24071	0.13977	0.0444	−0.0278	−0.11275
Average leptin relative copy number in ER+ tumors	0.15647 *	0.08372 *	0.01266 *	−0.04446	−0.2005
Average leptin relative copy number in ER^−^ tumors	0.65223 *	0.33081 *	0.14237 *	0.00842	−0.025

Linear regression analyses between FGFR1 copy number expression and relative leptin expression in breast cancer tumors from The Cancer Genome Atlas database demonstrate a significant correlation both in ER+ and ER^−^ breast tumor samples. * *p* < 0.05.

**Table 4:** Significant differences in average leptin expression between ER+ and ER^−^ breast tumor samples in FGFR1 amplification, FGFR1 gain, and FGFR1 diploid.

Breast Tumor FGFR1 Copy Number Status	FGFR1 Amplification	FGFR1 Gain	FGFR1 Diploid	FGFR1 Shallow Deletion	FGFR1 Deep Deletion
Standard deviation of ER+ Samples	0.2879	0.3001	0.3239	0.3152	0.2963
Standard deviation of ER- Samples	1.2884	0.2193	0.4196	0.5715	0.2524
*P*-value of one-sided t-test comparing average ER+ and ER- relative copy number	0.0025	0.0041	0.0003	0.1972	0.2668

Higher levels of average leptin copy number are present for ER^−^ tumors compared with ER^+^ tumors globally. Significant differences between ER^+^ and ER^−^ tumors were present between FGFR1 amplification, FGFR1 gain, and FGFR1 diploid breast tumors. Significant differences were observed between average relative leptin copy number corresponding between ER^+^ and ER^−^ tumors in FGFR1 amplification, FGFR1 gain and FGFR1 diploid categories. Student’s t-test was used to compare significant differences.
